# Determinants of intensive care unit light exposure: an observational cohort study of patient, temporal, and structural factors

**DOI:** 10.3389/fneur.2026.1821697

**Published:** 2026-08-24

**Authors:** Taylor A. Intihar, Omkara P. Rao, Preston A. Gomez-Crase, Margaret M. Doyle, Katherine Wasden, Kenneth P. Wright, Nancy S. Redeker, H. Klar Yaggi, Melissa P. Knauert

**Affiliations:** 1 Department of Internal Medicine, Yale University School of Medicine, New Haven, CT, United States; 2 Department of Integrative Physiology, University of Colorado Boulder, Boulder, CO, United States; 3 University of Connecticut Elisabeth DeLuca School of Nursing, Storrs, CT, United States

**Keywords:** circadian disruption, circadian rhythm, critical illness, ICU environment, intensive care unit, light exposure, lux, photometry

## Abstract

**Introduction:**

Patients with critical illness experience profound circadian disruption, driven in part by atypical light exposure in the intensive care unit (ICU). While prior studies characterize ICU light, the extent to which in patient, temporal, and structural factors determine light intensity remains unclear.

**Methods:**

We conducted an observational cohort study of light intensity in the rooms of patients admitted to one of thirteen medical ICU (MICU) rooms equipped with continuous light monitoring between February 2021 and 2022. Patients were included if they remained in the monitored MICU room for at least 48 h. We collected patient demographics and clinical features. We recorded light intensity (lux) every 60 s, and then calculated hourly mean lux, hourly proportion of measures ≥250 lux, and hourly proportion of measures ≤10 lux for the first 48 h of each patient’s MICU admission. Intraclass correlation coefficients (ICC) quantified variance in each light outcome attributable to patient-level factors. Multivariable multilevel models assessed adjusted associations between patient, temporal and structural factors and light outcomes. Both ICCs and multilevel models were generated separately for day (05:00–22:59) and night (23:00–04:59).

**Results:**

Among the 337 patient-room pairs, the mean (± standard deviation) age of patients was 66.1 ± 14.8 years; mean SOFA score was 6.8 ± 3.6; and daytime and nighttime mean lux were 173.8 ± 94.6 and 63.7 ± 88.2, respectively. ICCs indicated that patient-level factors explained 26.3% of daytime and 46.9% of nighttime variance in mean lux; ICC results were similar for other light outcomes. In multivariate models we found that temporal (e.g., time of day, season of ICU admission) and structural factors (e.g., room direction) strongly influenced daytime light levels, whereas nighttime variation was more heavily driven by patient-level factors such as mechanical ventilation status.

**Conclusion:**

ICU light exposure shows substantial between-patient variability, particularly at night. In multilevel models, we found that this variability is driven by clinical, temporal and structural factors. Our findings suggest that structural design alone is insufficient to normalize ICU light exposure, and that interventions targeting nocturnal care behaviors and daytime light provision may be necessary to mitigate circadian disruption in patients with critical illness.

## Introduction

The human circadian system is a fundamental homeostatic network that is frequently disrupted in patients with critical illness admitted to the intensive care unit (ICU). Circadian rhythms influence the function of essentially all cells, tissues, and organs in the body, thereby impacting far-reaching processes including sleep–wake, metabolism, cognition, cardiovascular function and immune function, among others. The central and peripheral clocks of the circadian system use cues, known as zeitgebers, to entrain these rhythms to a 24-h cycle that coincides with the earth’s day-night. It has been well established that light is the most influential zeitgeber (1) and that light patterns in the intensive care unit are abnormal (2–7).

To promote normal circadian alignment, light must have correct timing, duration, intensity, and spectra. Specifically, human circadian alignment is optimized in the presence of bright, full-spectrum daytime light that includes significant short-wavelength blue light and dim nighttime light that includes minimal short-wavelength blue light and less than 1 melanopic EDI lux (8–11). Existing studies of ICU light levels reveal a pervasive light pattern of “dim day, bright night” that does not meet the above metrics (2–7). Mean and median daytime light levels have been typically reported between 50 and 150 lux, rarely reaching 200 lux. Conversely, nighttime light levels are noted to frequently exceed the recommended limits and exhibit frequent peaks.

Abnormal day-night light patterns, such as those present in the ICU, can cause circadian disruption. Patients with critical illness are established to have circadian disruption, most commonly misalignment of a delayed type (12–15). In critical illness, circadian misalignment has been linked to decrements in cognition, alertness, mood, glucose control, cardiovascular function, and respiratory physiology (16, 17). Emerging evidence suggests that reversing abnormal ICU environmental cues may improve outcomes in critically ill patients. This is based primarily on the impact of multicomponent sleep-promotion bundles that seek to restore circadian alignment via improvements in day-night light patterns among other components of the bundle. These interventions have been associated with reductions in delirium and improvements in sleep (18).

To date, investigators have not robustly explored the associations between patient factors and ICU light intensity. There is limited evidence in our previously published work that light levels vary between occupied and unoccupied patient rooms (2), and that ventilator status impacts the use of bright lights at night (19). However, it remains unclear whether variation in patient light exposure is primarily attributable to structural or temporal factors, such as room orientation, daylight availability and time of day or attributable to patient-related factors such as illness severity that may influence care activities and artificial light use. Patients requiring more intensive monitoring, procedures, or bedside care may be exposed to greater illumination, particularly at night. Identifying these determinants may reveal modifiable targets for interventions designed to restore circadian-supportive light patterns and thus improve patient outcomes.

The objective of our study was to determine the relative contribution of patient, temporal, and structural factors to hourly light levels in a cohort of patients admitted to the medical ICU (MICU). We hypothesized that patient factors reflecting higher severity of illness (e.g., requiring mechanical ventilator, SOFA score) and logistic drivers of care (e.g., time of admission, isolation status) may be associated with the intensity of light levels during the ICU stay.

## Methods

### Ethics and reporting guidelines

Institutional Review Board approval was obtained with a waiver of consent (Yale HIC #2000038170). This study report was structured according to the STROBE checklist.

### Study design and setting

This was an observational cohort study conducted in two MICUs located on separate campuses of a single tertiary academic medical center. Both MICUs serve the same patient population, consist entirely of single-occupancy patient rooms with exterior windows, operate under the same protocols, share a common provider pool, and have around-the-clock intensivist staffing with nurse-to-patient ratios of 1:2 or 1:1. Additionally, all rooms have manually controlled fluorescent lighting, including overhead procedure lighting as well as multiple adjunct lights, including lights at the head of the bed and at counter workstations. The lights can generate a maximum brightness between 525 to 545 lux in full solar dark conditions with minor variation among rooms.

Thirteen of the 56 adult MICU rooms (8 of 41 on campus 1; 5 of 11 on campus 2) were selected for light measurements between February 2021 and 2022. Room selection considered head-of-bed orientation, the cardinal direction faced by the window, and the overall representativeness of the rooms with respect to the unit layout (see Supplementary Figure 1). We selected two rooms from each side of a rectangular floor plan on Campus 1; this included one room for each of the two possible bed orientations. Similarly, we selected two rooms each from two sides of a triangular floor plan on Campus 2. We selected an additional room on Campus 2 that uniquely had a bed that directly faced the window. We excluded one side of the triangular floor plan of Campus 2 due to variations in room configuration related to the installation and ongoing revision of temporary negative-pressure devices that were used for the Coronavirus Disease 2019 (COVID-19) pandemic response at our institution. Metered rooms from Campus 1 were located on the 9th floor, and metered rooms from Campus 2 were located on the 3rd floor of their respective buildings.

### Patient population

Patients were identified from an automated list of all MICU admissions during the study period. Patients were eligible for inclusion in the cohort if they were admitted to one of the light monitoring rooms during the 1-year observation period. Patients who were discharged from the MICU within 48 h of MICU admission (death or transfer), whose light monitored MICU admission was not the first ICU admission of the patient’s hospitalization, or who were co-enrolled in the active arm of a daytime bright light study were excluded.

A formal *a priori* sample size calculation was not performed. This observational cohort study included all eligible patients admitted to monitored ICU rooms during the one-year study period; therefore, the sample size was determined by the number of eligible patient-room pairs available during the study period.

### Light measures

Continuous light intensity (lux) was recorded every 60 s using the HOBO-OnSet MX2202 (LI-COR Environmental, Bourne, Massachusetts). This device is a factory-calibrated photopic light sensor; the device measures standard photopic illuminance (lux) over an operational range of 0–167,731 lux. The MX2202 device has a spectral sensitivity designed to approximate the photopic response of the human eye across the visible spectrum (i.e., 400–700 nm).

To approximate the angle of gaze, the light meter was mounted on the wall just above the head of the bed, without obstruction from the patient’s bed. The sensor placement was selected to minimize obstruction and support continuous light measurement as close as possible to the patient’s angle of gaze throughout the study period.

### Patient measures

Patient clinical data were collected from the electronic medical record, including demographics, MICU room assignment, date-time of hospital and MICU admission and discharge, mechanical ventilation (MV) status and date-time of intubation and extubation, COVID-19 status, isolation status, and clinical data to support the calculation of a Sequential Organ Failure Assessment (SOFA) score according to published protocols (20). Patient data were leveraged to calculate SOFA score, hospital and ICU length of stay, and length of intubation. Additionally, the electronic medical record was reviewed to establish 30-day survival regardless of hospital admission status.

### Statistical analysis

To allow for the likely difference in association between hypothesized predictors and light outcomes, separate analyses were performed for day and nighttime periods. In the analyses herein we defined day as 05:00–22:59 and night as 23:00–04:59; this reflected our wish to isolate night as a period defined by both full solar dark *and* reduced unit workflow. Outcomes examined included hourly mean lux, proportion of hourly measurements ≥250 lux and proportion of hourly measurements ≤10 lux during the first 48 h of each patient’s visit. The thresholds of ≥250 lux and ≤10 lux were selected *a priori* on the basis of contemporary circadian lighting recommendations indicating that daytime indoor light exposure should exceed 250 lux at the eye and that nighttime light exposure should remain below 10 lux to support circadian alignment and sleep (21, 22).

Hypothesized predictors included patient room direction, age, sex, SOFA score, mechanical ventilation during the 48-h light exposure period, ICU day (first 24 h versus second 24 h of 48-h light exposure period), ICU admission time (categorized in three-hour increments), COVID (SARS-CoV-2) infection status on admission, whether contact precautions were in place during the study period, seasonal quarter of ICU admission, and time interval of measurement. For night samples, time intervals were defined as the hour of the measurement. For day samples, time intervals were grouped into 3-h increments: 05:00–07:59, 08:00–10:59, 11:00–13:59, 14:00–16:59, 17:00–19:59, 20:00–22:59. Season centered quarter (seasonal quarter) of ICU admission was defined by centering each quarter on the respective equinox or solstice; this created the most homogenous within-quarter day lengths. The season centered quarters were as follows: spring equinox-centered quarter, February 4 to May 4; summer solstice-centered quarter, May 5 to August 5; fall equinox-centered quarter, August 6 to November 5; winter solstice-centered quarter, November 6 to February 3. Room direction was defined such that rooms facing the same cardinal direction on the same campus were grouped together in our analysis; direction groupings and overall unit structure are as indicated in Supplementary Figure 1.

We generated descriptive statistics for all hypothesized predictors and light outcomes. We then calculated intraclass correlation coefficients for each light outcome through a random intercepts model with patient identity as a random factor. Finally, a multivariate multilevel regression model with patient included as a random factor was generated for each outcome with all hypothesized predictors as independent variables. Adjusted outcome estimates derived from these models were plotted to allow for examination of the outcome across levels of key predictors. Statistical significance was set at *p* < 0.05 (two-tailed). Because these analyses were exploratory rather than confirmatory, no adjustment was made for multiple comparisons (23). All analyses were conducted in SAS 9.4 (SAS Institute Inc., Cary, NC).

## Results

A total of 337 patient-room pairs met the inclusion–exclusion criteria for this study. 317 patient-room pairs were excluded due to the patient staying less than 48 h (*n* = 262), non-first ICU admission (*n* = 53), and enrollment in a bright light study (*n* = 2). Patients had a mean age ± standard deviation (SD) of 66.1 ± 14.8 years and a mean SOFA score of 6.8 ± 3.6. Table 1 presents additional patient characteristics of the study cohort. Light was highly variable across day and night periods. There was a daytime mean lux of 173.8 ± 94.6 and a nighttime mean lux of 63.7 ± 88.2. Table 2 lists additional day and night light characteristics of the MICU.

**Table 1:** Patient characteristic; *N* = 337 patients-room pairs.

Patient characteristics	Value
Female sex: yes, percentage	49.9%
Age: years, mean ± SD	66.1 ± 14.8
SOFA score: mean ± SD	6.8 ± 3.6
ICU length of stay: days, mean ± SD	6.7 ± 6.1
Hospital length of stay: days, mean ± SD	23.0 ± 38.5
Mechanical ventilation status: yes, percentage	43.3%
COVID on admission to hospital: yes, percentage	14.8%
Contact precautions: yes, percentage	48.7%
30-day mortality: yes, percentage	30.0%

COVID, SARS-Cov-2; ICU, intensive care unit; SD, standard deviation; SOFA, Sequential Organ Failure Assessment.

**Table 2:** Light characteristic; *N* = 337 patients-room pairs.

Light characteristics	Mean ± SD
Daytime (05:00–22:59)	
Mean lux	173.8 ± 94.6
Proportion ≥250 lux	0.28 ± 0.23
proportion ≤10 lux	0.16 ± 0.11
Nighttime (23:00–04:59)	
Mean lux	63.7 ± 88.2
Proportion ≥250 lux	0.10 ± 0.21
proportion ≤10 lux	0.62 ± 0.34

SD, standard deviation.

Intraclass correlation analysis revealed that the proportion of variance in mean lux levels attributable to patient-level factors was 26.3 and 46.9% for day and night, respectively (Table 3); the proportions of variance attributable to patient-level factors were similar when examining the proportion of readings ≥250 lux. In analyses of the proportion of readings ≤10 lux, a measure of low light, variance attributable to patient-level factors was 9.1 and 54.1% for the day and night, respectively.

**Table 3:** Intraclass correlation coefficient (ICC) variance due to patient factors.

Measure of light	Percentage explained by individual factors
Daytime (05:00–22:59)	
Mean lux	26.3%
Proportion ≥ 250 lux	27.3%
Proportion ≤ 10 lux	9.1%
Nighttime (23:00–04:59)	
Mean lux	46.9%
Proportion ≥ 250 lux	49.6%
Proportion ≤ 10 lux	54.1%

Table 4 presents findings for multivariate multilevel regression models of mean lux. Detailed listings of adjusted estimated means of the modeled outcomes by key predictors are provided in Supplementary Table 1 and Supplementary Figures 2, 3. Among the tested demographic and clinical factors, SOFA scores, ventilation status, contact precaution status, day of ICU admission, and time of ICU admission were significantly associated with mean light levels during the day and/or night. Higher SOFA scores were associated with higher mean light levels at night (*β*-coefficient 2.73, standard error (SE) 1.35, *p* = 0.042). Mechanically ventilated patients had higher mean light levels during the day (*β*-coefficient 26.63, SE 10.93, *p* = 0.015) and night (β-coefficient 26.41, SE 9.82, *p* = 0.007). Contact precautions decreased mean light levels during the day (β-coefficient −25.61, SE 10.09, *p* = 0.011).

**Table 4:** Multivariate multilevel regression model for mean light intensity (lux) during daytime (05:00–22:59) and nighttime (23:00–04:59) periods with patient as a random factor.

A. Categorical patient factors						
	Daytime Mean Lux			Nighttime Mean Lux		
	β coeff	St error	*p*-value	β coeff	St error	*p*-value
Intercept	78.89	(32.56)	.	102.86	(29.29)	.
Age (per 1 year)	0.04	(0.34)	0.911	−0.48	(0.31)	0.117
Female sex	−1.12	(9.99)	0.911	12.14	(8.98)	0.176
SOFA (per 1 point)	2.19	(1.50)	0.144	2.73	(1.35)	0.042
Mechanical vent	26.63	(10.93)	0.015	26.41	(9.82)	0.007
COVID positive	12.89	(15.68)	0.411	12.28	(14.05)	0.382
Contact precautions	−25.61	(10.09)	0.011	−16.30	(9.06)	0.072
Day 1 of ICU admit	30.07	(2.58)	<0.0005	25.68	(2.71)	<0.0005
Time of ICU admission, referenced to 08:00–10:59						
05:00–07:59	39.50	(22.17)	0.561	−2.34	(19.91)	0.050
11:00–13:59	16.94	(23.02)		−22.97	(20.67)	
14:00–16:59	−5.40	(20.28)		−12.72	(18.21)	
17:00–19:59	16.95	(20.19)		24.55	(18.13)	
20:00–22:59	6.94	(18.86)		23.23	(16.94)	
23:00–01:59	8.94	(20.54)		22.60	(18.45)	
02:00–04:59	15.35	(20.74)		9.17	(18.63)	
Season centered quarters, referenced to fall centered quarter (August 6 to November 5)						
Winter	−34.94	(14.52)	0.062	−7.76	(12.66)	0.507
Spring	−12.83	(13.37)		−13.60	(12.06)	
Summer	0.00	(12.74)		−16.82	(11.56)	
Cardinal direction, referenced to ‘direction 1’—campus 1, southwest facing rooms						
Direction 2, C1, NW	24.45	(17.00)	<0.0005	0.26	(15.27)	<0.0005
Direction 3, C1, NE	−2.79	(18.19)		−42.48	(16.34)	
Direction 4, C1, SE	34.66	(19.20)		−23.18	(17.24)	
Direction 5, C2, SE	−64.89	(17.37)		−63.23	(15.61)	
Direction 6, C2, SE	−27.41	(21.10)		−64.80	(18.95)	
Direction 7, C2, NE	−18.04	(17.72)		−58.39	(15.92)	

B. Time of day, referenced to 05:00–07:59 for day and 23:00–23:59 for night							
Daytime mean lux				Nighttime mean lux			
Time of day	β coeff	St error	*p*-value	Time of night	β coeff	St error	*p*-value
08:00–10:59	129.07	(4.48)	<0.0005	00:00–00:59	−12.05	(4.70)	<0.0005
11:00–13:59	125.54	(4.46)		01:00–01:59	−18.91	(4.70)	
14:00–16:59	98.32	(4.46)		02:00–02:59	−20.94	(4.70)	
17:00–19:59	59.50	(4.46)		03:00–03:59	−20.32	(4.70)	
20:00–22:59	7.97	(4.46)		04:00–04:59	−15.77	(4.70)	

Seasonal quarters are time periods centered around the spring and fall equinox and the winter and summer solstice for the period of light monitoring. Significant *p*-values are highlighted via green fill. β Coeff, Beta-coefficient; C1, campus 1; C2, campus 2; COVID, SARS-Cov-2, ICU; Day 1 of ICU Admit, first 24 out of 48 h of MICU admission; intensive care unit; mechanical vent, mechanical ventilation; NE, northeast; NW, northwest; SE, southeast; SOFA, Sequential Organ Failure Assessment; St Error, standard error.

MICU Day 1, the first 24 h of MICU admission, was associated with higher mean light levels during day (*β*-coefficient 30.07, SE 2.58, *p* < 0.0005) and night (β-coefficient 25.68, SE 2.71, p <0.0005). Importantly, patients admitted earlier in the day, had significantly (*p* = 0.05) lower mean light levels at night; for example, patients admitted between 20:00–22:59 had a *β*-coefficient of 23.23, SE 16.94 versus the reference period of 08:00–10:59. Corresponding analyses for the models of proportion of measurements ≥250 and ≤10 lux are presented in Supplementary Tables 2, 3.

As might be expected, time of day significantly impacted light levels in all models. Using a reference period of 05:00–07:59, we established that time periods 08:00–10:59 (*β*-coefficient 129.08, SE 4.48), and 11:00–13:59 (β-coefficient 125.5, SE 4.46) were associated with the highest mean light levels; mean lux steadily decreases during the remaining daytime periods. Overnight periods, referenced to the 23:00–23:59 h, had β-coefficients that varied between −12.05 and −20.94 lux (SE 4.70, *p* < 0.0005). These results were largely recapitulated in analyses of the proportion of measurements ≥250 and ≤10 lux. Season of admission was marginally (*p* = 0.062) associated with daytime mean light levels.

Finally, room direction had a significant impact on light levels across both day and night periods and for all light outcomes. In comparison to the southwest-facing rooms on campus 1 (direction 1, reference), mean lux *β*-coefficients varied between −64.89 and 34.66 with the highest daytime light levels occurring in ‘direction 4’, the southeast-facing rooms of campus 1. The estimated proportion of measurements ≥250 was also highest for this direction.

## Discussion

To our knowledge, this is among the first studies to explore associations between individual patient factors and changes in ICU light intensity. We assessed light outcomes in the first 48 h of MICU admission and demonstrated significant variation in light due to several key patient factors. For example, we observed that patients admitted in the evening and overnight periods and patients requiring mechanical ventilation experience higher mean levels of light across day and night periods. Conversely, patients assigned contact precautions are exposed to lower light intensity. These insights immediately inform the design of sleep, circadian, and delirium interventions in the ICU. Furthermore, we note significant differences in light exposure by room direction, with nearly 100 lux differences in daytime mean light intensity between the brightest and dimmest directions.

Overall, we found day and night light intensities in our ICU were consistent with prior work in this domain (2–7). Mean daytime light levels fell below 250 lux, a minimal standard intensity set forth in expert guidelines (21, 22); the proportion of time ≥250 was assessed for this reason. While just over one quarter of daytime readings met or exceeded 250 lux, this distribution was not uniform across all room directions, with some receiving far more and others receiving far less. Conversely, nighttime mean light levels averaged 60 lux, again with a high degree of variability despite our highly restrictive definition of night from 23:00 to 04:59. These nighttime light levels exceeded the recommended limit of 0 to 10 lux more than one-third of the time, reinforcing the prior work reporting that daytime is too dim and nighttime is too bright in the ICU.

Intraclass correlation coefficients assessing the proportion of total variation in the light outcomes demonstrated that differences between patients account for a sizable proportion of variance, particularly during the night. We hypothesize that during the day, natural sunlight at least partially overrides the contribution of artificial light to a room’s lux level and obscures the contribution of patient factors to the overall light intensity. Beyond day-night differences, the significant changes associated with time of ICU admission and day 1 of MICU admission (versus day 2) suggest that increases in light levels are associated with increases in clinical care that occur disproportionately at the start of ICU admission. Similarly, patients who were mechanically ventilated had both higher mean nighttime and daytime lux. Prior work in our group (19) demonstrated that a sleep promotion intervention targeting light use resulted in significantly decreased bright light use at night in patients requiring mechanical ventilation but no change for patients without mechanical ventilation. This suggests that both staff care habits *and* patient preferences may contribute to room light levels and brings into question whether the higher light levels observed in the current study are truly needed for effective care. Furthering this idea are the lower light levels observed in patients with contact precaution restrictions in place.

In assessing the impact of structural and temporal factors on light intensity, we identified meaningful variability in light measurements across room directions and time-of-day intervals. Rooms on Campus 1 sustained a higher mean daytime lux and a greater proportion of readings ≥250 versus Campus 2, possibly attributable to the lower elevation of the rooms on Campus 2 (3rd floor versus 9th floor on Campus 1) and their relatively greater risk of solar obstruction from surrounding infrastructure. Overnight light levels also varied significantly based on room direction, suggesting that hallway lighting and external, artificial nighttime light sources (e.g., parking garages, local buildings with bright lighting) do influence room light intensity. Evaluation of light levels by time of day showed a rise and fall in mean daytime lux that reflected the progression of the solar day. Peak mean lux occurred between the morning and early afternoon, suggesting that solar influence remains a primary driver of light exposure during the period critical for circadian alignment. Though light intensity is dimmer at night, lux levels remain well above recommended limits.

The diverse sources of light variation in ICU rooms have direct implications for the design of circadian countermeasures in the ICU. The important contributions of patient factors suggest that architectural solutions alone (e.g., larger windows or circadian-tuned lighting systems) may not overcome abnormal ICU lighting patterns. Additionally, even rooms with optimal orientation and location do not achieve sufficiently high daytime light levels, suggesting the necessity of a multimodal approach. To reduce nocturnal light, it is likely important that we separate nighttime “care delivery” from “overhead lighting” (e.g., using task lighting). Furthermore, additional daytime full-spectrum light sources with sufficient intensity may be needed to promote normal circadian alignment in patients with critical illness.

## Limitations

This was a single-center, observational study with retrospective patient data collection; therefore, patient data was limited to data available in the electronic medical record. There are likely additional patient factors associated with variation in light intensity, notably patient, staff, and visitor preferences. Further, to prioritize feasibility and enable long-term, continuous monitoring, we selected lux intensity rather than the melanopic index for this longitudinal light-monitoring project. Additional studies of melanopic index in ICU rooms would be of value but were outside the scope of this project. Finally, our light measures were positioned above the head of the bed as the best approximation for angle of gaze; we believe this to be the best approximation possible during this prolonged monitoring period.

## Conclusion

Here we demonstrate that patient factors accounted for a substantial proportion of the variation in MICU light levels, particularly during the nocturnal period. Our findings suggest that the drivers of this variation shift depending on the time of day. During daytime hours, light exposure appears largely driven by temporal and structural factors. However, at night, we see increased association between light variation and patient factors such as those related to the timing of ICU admission and mechanical ventilation status. Nonetheless, the extent to which the observed light exposure reflects clinical necessity rather than behavioral habits or local unit culture remains unclear. Therefore, future studies should aim to characterize these behavioral forces by expanding the analysis to include unit-level behavioral factors, such as visitor presence, nursing intervention frequency, and provider-specific workflow patterns.

## Data Availability

The raw data supporting the conclusions of this article will be made available by the authors, without undue reservation.
