# Supplementary material for: Determinants of intensive care unit light exposure: an observational cohort study of patient, temporal, and structural factors
Source: Front Neurol. 2026 Aug 24;17:1821697. doi: 10.3389/fneur.2026.1821697 (PMC13547019; doi:10.3389/fneur.2026.1821697)
Supplement: Supplementary file 1 [file Data_Sheet_1.PDF]

Determinants of ICU Light Exposure
Intihar et al.

Supplementary Figure 1: Medical Intensive Care Unit floorplan. (A) Campus 1 which has 28 beds on one floor and an additional 13 beds on an adjacent floor; all monitored beds were on a single floor of this campus (9th floor). (B) Campus 2 which has 16 beds on a single floor (3rd floor). All rooms have an exterior window; the exterior window location and directionality coincides with the text indicating 'Direction' in all cases. Green fill indicates light monitoring room; grey fill indicates non-patient-occupied spaces (e.g., storage, workstations); filled rectangles (■) indicate patient head of bed; arrows indicate entrance/exit of unit.

A.

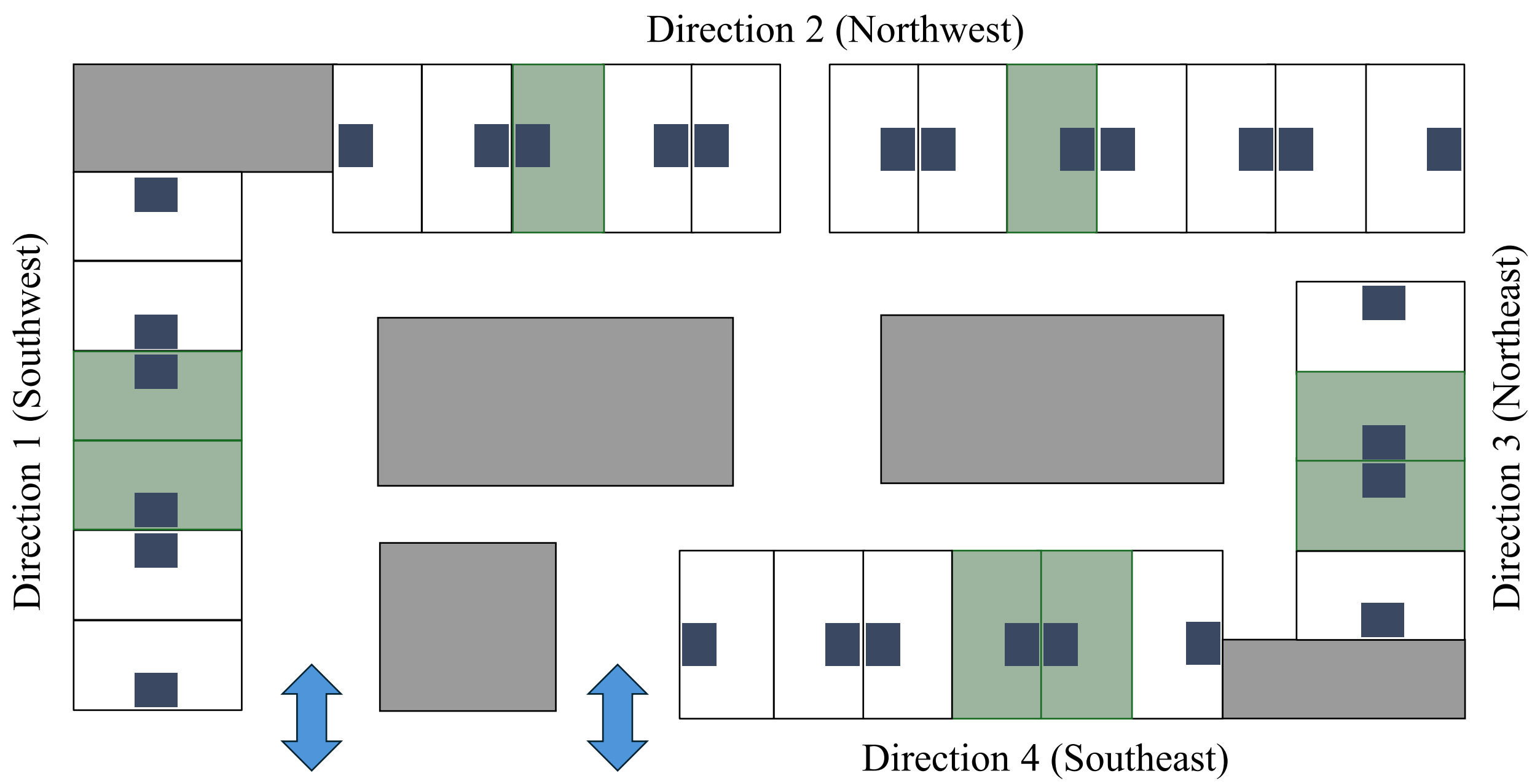

B.

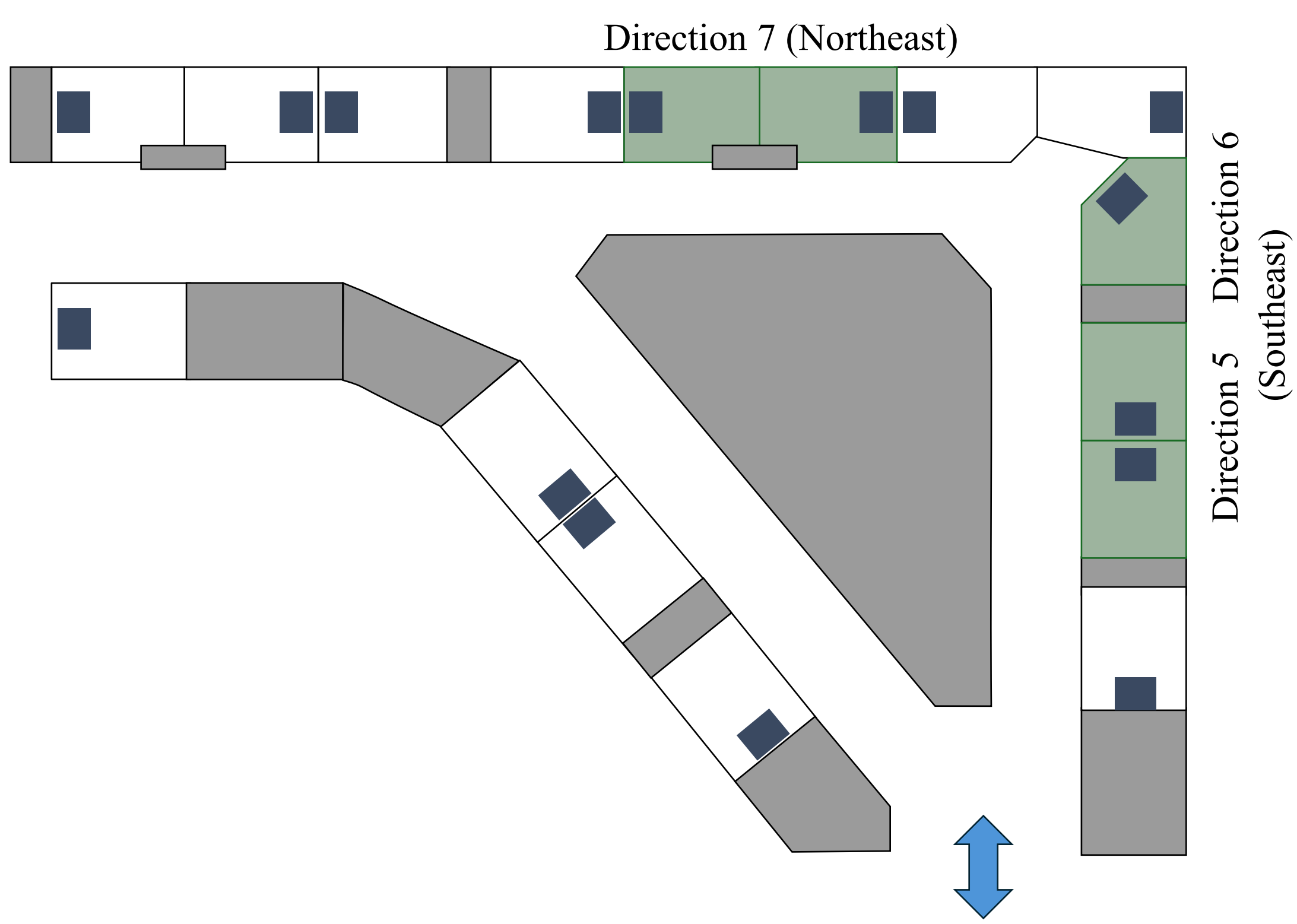

Supplementary Table 1A: Adjusted outcome estimates and 95% confidence intervals by time of day.

	Mean Lux (95% CI)	Proportion ≥250 (95% CI)	Proportion ≤10 (95% CI)
**Daytime Light Levels by 3-hour Period**			
05:00 – 07:59	111 (95, 126)	0.16 (0.12, 0.19)	0.33 (0.31, 0.35)
08:00 - 10:59	240 (224, 255)	0.38 (0.34, 0.41)	-0.00 (-0.02, 0.01)
11:00 – 13:59	236 (221, 251)	0.40 (0.36, 0.43)	-0.01 (-0.03, 0.01)
14:00 – 16:59	209 (194, 224)	0.34 (0.30, 0.37)	0.01 (-0.00, 0.03)
17:00 – 19:59	170 (155, 185)	0.26 (0.23, 0.30)	0.14 (0.12, 0.15)
20:00 – 22:59	119 (103, 134)	0.19 (0.15, 0.22)	0.39 (0.37, 0.41)
**Nighttime Light Levels by Hour Period**			
23:00 – 23:59	79 (65, 93)	0.12 (0.09, 0.16)	0.52 (0.47, 0.57)
00:00 – 00:59	67 (53, 81)	0.11 (0.07, 0.14)	0.57 (0.52, 0.62)
01:00 – 01:59	60 (46, 74)	0.09 (0.06, 0.13)	0.61 (0.56, 0.66)
02:00 – 02:59	58 (44, 72)	0.09 (0.05, 0.12)	0.61 (0.56, 0.66)
03:00 – 03:59	59 (45, 73)	0.09 (0.06, 0.13)	0.61 (0.56, 0.66)
04:00 – 04:59	64 (50, 77)	0.10 (0.06, 0.13)	0.59 (0.54, 0.64)

Abbreviations: CI, confidence interval; Proportion ≥250, proportion of readings greater than 250 lux; Proportion ≤10, proportion of all readings less than or equal to 10 lux.

Supplementary Table 1B: Adjusted outcome estimates and 95% confidence intervals by patient characteristics, season of admission and room direction.

	Mean Lux (95% CI)	
	**Day**	**Night**
**Light Levels by Patient Characteristic**		
COVID (-)	174 (163, 186)	58 (48, 69)
COVID (+)	187 (160, 214)	71 (46, 95)
Male sex	181 (165, 198)	59 (44, 73)
Female sex	180 (162, 198)	71 (55, 87)
Contact (-)	193 (176, 211)	73 (57, 88)
Contact (+)	168 (151, 185)	56 (41, 72)
Day 1 of ICU Admit	196 (181, 210)	77 (65, 90)
Day 2 of ICU Admit	166 (151, 180)	52 (39, 65)
Ventilated (-)	167 (150, 185)	51 (36, 67)
Ventilated (+)	194 (176, 212)	78 (62, 94)
**Light Levels by Time of ICU Admission**		
02:00 – 04:59	184 (155, 212)	69 (43, 94)
05:00 – 07:59	208 (175, 241)	57 (28, 87)
08:00 - 10:59	168 (136, 200)	59 (31, 88)
11:00 – 13:59	185 (151, 219)	36 (6, 67)
14:00 – 16:59	163 (135, 190)	47 (22, 71)
17:00 – 19:59	185 (157, 213)	84 (59, 109)
20:00 – 22:59	175 (150, 200)	83 (60, 105)
23:00 – 01:59	177 (149, 206)	82 (57, 108)
**Light Levels by Season Centered Quarters**		
Fall	193 (171, 215)	74 (55, 94)
Spring	180 (157, 202)	61 (40, 81)
Summer	193 (171, 214)	57 (38, 77)
Winter	158 (137, 178)	66 (48, 85)
**Light Levels by Room Direction**		
Direction 1, C1, SW	188 (160, 216)	101 (75, 126)
Direction 2, C1, NW	213 (188, 238)	101 (78, 124)
Direction 3, C1, NE	186 (157, 214)	58 (33, 84)
Direction 4, C1, SE	223 (191, 255)	77 (49, 106)
Direction 5, C2, SE	123 (99, 148)	37 (15, 60)
Direction 6, C2, SE	161 (129, 193)	36 (7, 64)
Direction 7, C2, NE	170 (145, 195)	42 (20, 64)

Abbreviations: C1, campus 1; C2, campus 2; CI, confidence interval; COVID, SARS-Cov-2; Day 1 of ICU Admit, first 24 out of 48 hours of MICU admission; NE, northeast; NW, northwest; Proportion ≥250, proportion of readings greater than 250 lux; Proportion ≤10, proportion of all readings less than or equal to 10 lux; SE, southeast; SW, southwest.

Supplementary Table 1C: Adjusted outcome estimates and 95% confidence intervals by patient characteristics, season of admission and room direction.

	Proportion ≥250 (95% CI)		Proportion ≤10 (95% CI)	
	**Day**	**Night**	**Day**	**Night**
**Light Levels by Patient Characteristics**				
COVID (-)	0.27 (0.25, 0.30)	0.09 (0.06, 0.11)	0.16 (0.15, 0.17)	0.64 (0.61, 0.68)
COVID (+)	0.30 (0.24, 0.36)	0.11 (0.05, 0.17)	0.13 (0.10, 0.15)	0.53 (0.44, 0.62)
Male sex	0.29 (0.25, 0.33)	0.09 (0.05, 0.12)	0.14 (0.13, 0.16)	0.61 (0.56, 0.66)
Female sex	0.29 (0.24, 0.33)	0.12 (0.08, 0.15)	0.14 (0.12, 0.16)	0.56 (0.51, 0.62)
Contact (-)	0.32 (0.28, 0.36)	0.12 (0.09, 0.16)	0.14 (0.12, 0.16)	0.57 (0.51, 0.62)
Contact (+)	0.25 (0.21, 0.29)	0.08 (0.04, 0.12)	0.15 (0.13, 0.17)	0.61 (0.55, 0.66)
Day 1 of ICU Admit	0.31 (0.28, 0.34)	0.11 (0.08, 0.15)	0.13 (0.11, 0.14)	0.53 (0.49, 0.58)
Day 2 of ICU Admit	0.26 (0.23, 0.30)	0.09 (0.06, 0.12)	0.16 (0.15, 0.18)	0.64 (0.59, 0.69)
Ventilated (-)	0.27 (0.23, 0.31)	0.09 (0.05, 0.13)	0.17 (0.15, 0.19)	0.68 (0.62, 0.73)
Ventilated (+)	0.30 (0.26, 0.34)	0.11 (0.07, 0.15)	0.12 (0.10, 0.13)	0.49 (0.44, 0.55)
**Light Levels by Time of ICU Admission**				
02:00 – 04:59	0.29 (0.22, 0.36)	0.11 (0.05, 0.17)	0.12 (0.09, 0.15)	0.55 (0.45, 0.64)
05:00 – 07:59	0.30 (0.22, 0.38)	0.07 (0.00, 0.15)	0.12 (0.09, 0.16)	0.64 (0.53, 0.75)
08:00 - 10:59	0.28 (0.20, 0.35)	0.12 (0.05, 0.19)	0.17 (0.14, 0.20)	0.64 (0.54, 0.75)
11:00 – 13:59	0.30 (0.22, 0.38)	0.04 (-0.04, 0.11)	0.15 (0.11, 0.18)	0.72 (0.61, 0.83)
14:00 – 16:59	0.26 (0.20, 0.33)	0.06 (-0.00, 0.12)	0.15 (0.12, 0.17)	0.61 (0.52, 0.70)
17:00 – 19:59	0.30 (0.23, 0.37)	0.15 (0.09, 0.22)	0.14 (0.11, 0.17)	0.51 (0.42, 0.60)
20:00 – 22:59	0.28 (0.22, 0.34)	0.13 (0.07, 0.18)	0.14 (0.12, 0.17)	0.53 (0.45, 0.61)
23:00 – 01:59	0.28 (0.21, 0.35)	0.13 (0.06, 0.19)	0.15 (0.13, 0.18)	0.49 (0.40, 0.58)
**Light Levels by Season Centered Quarters**				
Fall	0.31 (0.26, 0.37)	0.14 (0.09, 0.18)	0.15 (0.13, 0.17)	0.60 (0.53, 0.67)
Spring	0.29 (0.24, 0.35)	0.09 (0.04, 0.14)	0.14 (0.11, 0.16)	0.55 (0.48, 0.63)
Summer	0.30 (0.25, 0.35)	0.09 (0.04, 0.14)	0.11 (0.09, 0.13)	0.60 (0.53, 0.67)
Winter	0.23 (0.19, 0.28)	0.09 (0.05, 0.14)	0.17 (0.15, 0.19)	0.60 (0.53, 0.66)
**Light Levels by Room Direction**				
Direction 1, C1, SW	0.34 (0.27, 0.40)	0.16 (0.10, 0.22)	0.11 (0.08, 0.13)	0.47 (0.38, 0.56)
Direction 2, C1, NW	0.40 (0.34, 0.46)	0.20 (0.14, 0.25)	0.07 (0.05, 0.10)	0.37 (0.29, 0.45)
Direction 3, C1, NE	0.29 (0.23, 0.36)	0.10 (0.04, 0.16)	0.09 (0.06, 0.12)	0.47 (0.38, 0.56)
Direction 4, C1, SE	0.40 (0.33, 0.48)	0.15 (0.08, 0.22)	0.15 (0.11, 0.18)	0.64 (0.54, 0.75)
Direction 5, C2, SE	0.13 (0.07, 0.19)	0.04 (-0.01, 0.10)	0.22 (0.19, 0.24)	0.74 (0.66, 0.82)
Direction 6, C2, SE	0.20 (0.12, 0.27)	0.04 (-0.03, 0.11)	0.16 (0.13, 0.20)	0.65 (0.55, 0.76)
Direction 7, C2, NE	0.24 (0.18, 0.30)	0.01 (-0.04, 0.07)	0.20 (0.18, 0.23)	0.76 (0.68, 0.84)

Abbreviations: C1, campus 1; C2, campus 2; CI, confidence interval; COVID, SARS-Cov-2; Day 1of ICU Admit, first 24 out of 48 hours of MICU admission; NE, northeast; NW, northwest; Proportion ≥250, proportion of readings greater than or equal to 250 lux; Proportion ≤10, proportion of all readings less than or equal to 10 lux; SE, southeast; SW, southwest.

Supplementary Figure 2: Light levels by time of day. Filled circle (●) indicates adjusted outcome estimates; whiskers (⊢┤) indicate 95% confidence interval.(A) Mean lux during day periods; (B) Mean lux during night periods; (C) Proportion of light ≥250 lux during day periods; (D) Proportion of light ≥250 lux during night periods; (E) Proportion of light ≤10 lux during day periods; (F) Proportion of light ≤10 lux during night periods.

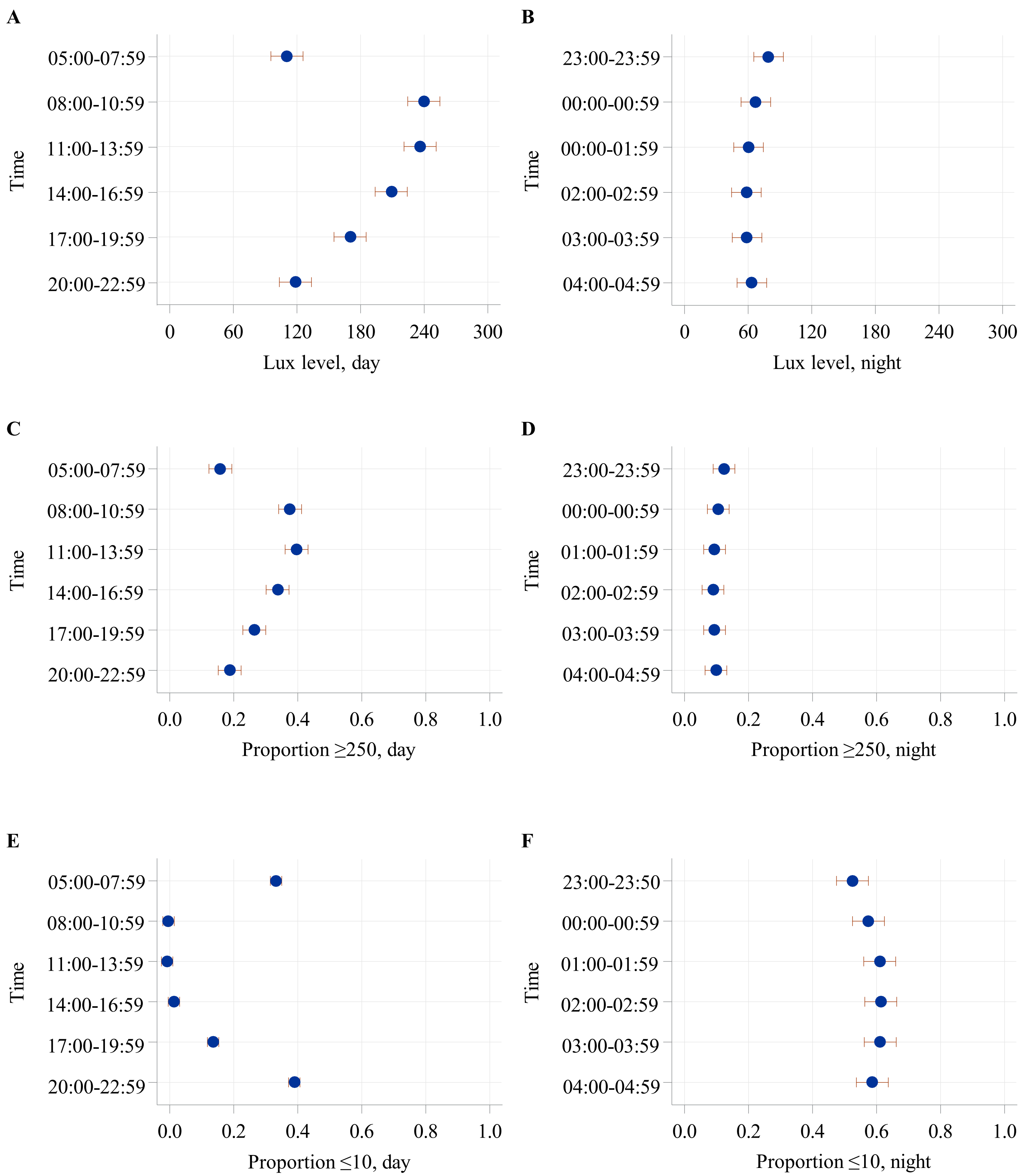

Supplementary Figure 3: Light levels by direction. Filled circle (●) indicates adjusted outcome estimates; whiskers (—|) indicate 95% confidence interval. (A) Mean lux by direction during the day; (B) Mean lux by direction during the night; (C) Proportion of light ≥250 lux by direction during the day; (D) Proportion of light ≥250 lux by direction during the night; (E) Proportion of light ≤10 lux by direction during the day; (F) Proportion of light ≤10 lux by direction during the day. Direction abbreviations: D1, SW, direction 1 on campus 1 facing southwest; D2,NW, direction 2 on campus 1 facing northwest; D3, NE, direction 3 on campus 1 facing northeast; D4,SE, direction 4 on campus 1 facing southeast; D5,SE, direction 5 on campus 2 facing southeast; D6,SE, direction 6 on campus 2 facing southeast with bed facing window; D7,NE, direction 7 on campus 2 facing northeast.

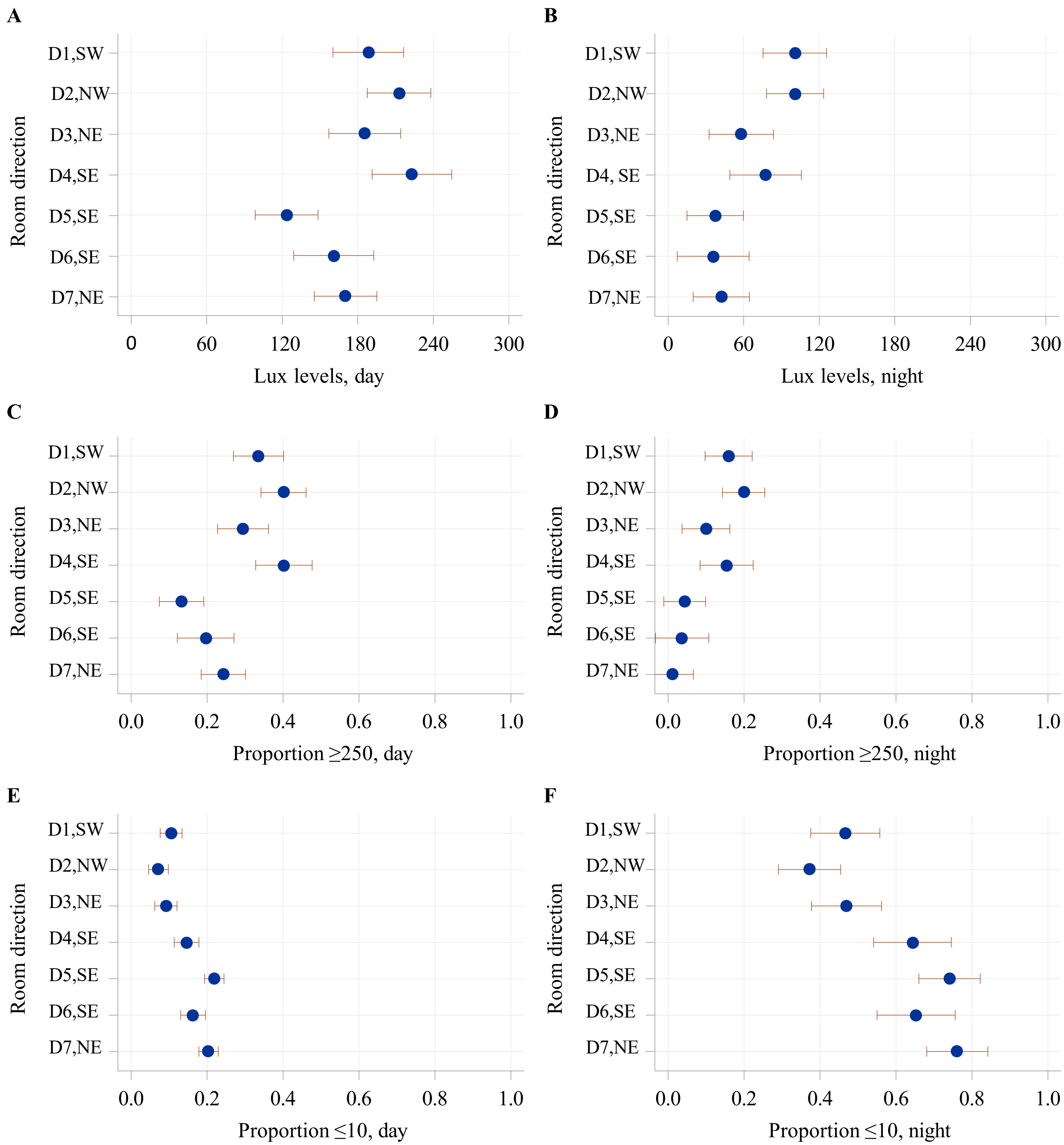

Supplementary Table 2: Multivariate multilevel regression model for Proportion ≥250 Lux during daytime (05:00 – 22:59) and nighttime (23:00-04:59) periods with patient as random factor. Note seasonal centered quarters are time periods centered around the spring and fall equinox and the winter and summer solstice for the period of light monitoring. Significant p-values are highlighted via green fill.

A. Categorical patient factors.

	Daytime Proportion ≥250 Lux			Nighttime Proportion ≥250 Lux		
	β Coeff	St Error	p-value	β Coeff	St Error	p-value
**Intercept**	**0.184**	**(0.077)**		**0.259**	**(0.072)**	
Age (per 1 year)	0.000	(0.001)	0.913	-0.001	(0.001)	0.148
Female Sex	-0.001	(0.023)	0.960	0.030	(0.022)	0.172
SOFA (per 1 point)	0.004	(0.004)	0.235	0.003	(0.003)	0.305
Mechanical Vent	0.031	(0.026)	0.234	0.021	(0.024)	0.388
COVID Positive	0.026	(0.037)	0.485	0.023	(0.035)	0.500
Contact Precautions	-0.068	(0.024)	0.004	-0.046	(0.022)	0.038
Day 1 of ICU Admit	0.049	(0.006)	0.000	0.026	(0.006)	0.000
**Time of ICU Admission, Referenced to 08:00-10:59**						
05:00 – 07:59	0.024	(0.052)		-0.045	(0.049)	
11:00 – 13:59	0.028	(0.054)		-0.085	(0.051)	
14:00 – 16:59	-0.015	(0.048)		-0.061	(0.045)	
17:00 – 19:59	0.024	(0.047)	0.986	0.034	(0.045)	0.134
20:00 – 22:59	0.003	(0.044)		0.008	(0.042)	
23:00 – 01:59	0.003	(0.048)		0.006	(0.045)	
02:00 – 04:59	0.013	(0.049)		-0.012	(0.046)	
**Season Centered Quarter, Referenced to Fall Centered Quarter**						
Winter	-0.079	(0.034)		-0.045	(0.031)	0.261
Spring	-0.020	(0.031)	0.119	-0.051	(0.030)	
Summer	-0.012	(0.030)		-0.045	(0.028)	
**Cardinal Direction, Referenced to 'Direction 1' - Campus 1, Southwest Facing Rooms**						
Direction 2, C1, NW	0.066	(0.040)		0.040	(0.038)	
Direction 3, C1, NE	-0.041	-0.043		-0.060	(0.040)	
Direction 4, C1, SE	0.067	(0.045)	<0.0005	-0.005	(0.042)	<0.0005
Direction 5, C2, SE	-0.203	(0.041)		-0.116	(0.038)	
Direction 6, C2, SE	-0.139	(0.050)		-0.122	(0.047)	
Direction 7, C2, NE	-0.093	(0.042)		-0.148	(0.039)	

B. Time of Day, Referenced to 05:00-07:59 for Day and 23:00-23:59 for Night

Daytime Proportion ≥250 Lux				Nighttime Proportion ≥250 Lux			
Time of Day	β Coeff	St Error	p-value	Time of Night	β Coeff	St Error	p-value
08:00 - 10:59	0.218	(0.011)		00:00 – 00:59	-0.018	(0.011)	
11:00 – 13:59	0.238	(0.011)		01:00 – 01:59	-0.029	(0.011)	
14:00 – 16:59	0.179	(0.011)	<0.0005	02:00 – 02:59	-0.035	(0.011)	0.023
17:00 – 19:59	0.106	(0.011)		03:00 – 03:59	-0.029	(0.011)	
20:00 – 22:59	0.029	(0.011)		04:00 – 04:59	-0.025	(0.011)	

Abbreviations: β Coeff, Beta-coefficient; C1, campus 1; C2, campus 2; COVID, SARS-CoV-2; Day 1, first 24 hours of 48 hour monitoring period; ICU, intensive care unit; Mechanical Vent, Mechanical Ventilation; NE, northeast; NW, northwest; Proportion ≥250, proportion of readings greater than or equal to 250 lux; SE, southeast; SOFA, Sequential Organ Failure Assessment; St Error, standard error.

Supplementary Table 3: Multivariate multilevel regression model for Proportion ≤10 Lux during daytime (05:00 – 22:59) and nighttime (23:00-04:59) periods with patient as random factor. Note seasonal centered quarters are time periods centered around the spring and fall equinox and the winter and summer solstice for the period of light monitoring. Significant p-values are highlighted via green fill.

A. Categorical patient factors.

	Daytime Proportion ≤10 Lux			Nighttime Proportion ≤10 Lux		
	β Coeff	St Error	p-value	β Coeff	St Error	p-value
**Intercept**	0.425	(0.034)		0.724	(0.106)	
Age (per 1 year)	0.000	(0.000)	0.898	0.001	(0.001)	0.501
Female Sex	-0.002	(0.010)	0.815	-0.047	(0.032)	0.143
SOFA (per 1 point)	-0.005	(0.002)	0.001	-0.014	(0.005)	0.005
Mechanical Vent	-0.056	(0.011)	<0.0005	-0.184	(0.035)	<0.0005
COVID Positive	-0.034	(0.016)	0.034	-0.115	(0.051)	0.023
Contact Precautions	0.011	(0.010)	0.301	0.040	(0.033)	0.223
Day 1 of ICU Admit	-0.035	(0.005)	<0.0005	-0.105	(0.009)	<0.0005
**Time of ICU Admission, Referenced to 08:00-10:59**						
05:00 – 07:59	-0.047	(0.023)		-0.005	(0.072)	
11:00 – 13:59	-0.021	(0.024)		0.076	(0.075)	
14:00 – 16:59	-0.024	(0.021)		-0.031	(0.066)	
17:00 – 19:59	-0.03	(0.021)	0.262	-0.134	(0.065)	0.006
20:00 – 22:59	-0.029	(0.019)		-0.118	(0.061)	
23:00 – 01:59	-0.016	(0.021)		-0.154	(0.067)	
02:00 – 04:59	-0.053	(0.021)		-0.099	(0.067)	
**Season Centered Quarter, Referenced to Fall Centered Quarter (August 6 to November 5)**						
Winter	0.022	(0.015)		-0.003	(0.045)	
Spring	-0.012	(0.014)	<0.0005	-0.045	(0.043)	0.674
Summer	-0.040	(0.014)		0.000	(0.042)	
**Cardinal Direction, Referenced to 'Direction 1' - Campus 1, Southwest Facing Rooms**						
Direction 2, C1, NW	-0.033	(0.017)		-0.094	(0.055)	
Direction 3, C1, NE	-0.014	(0.019)		0.003	(0.059)	
Direction 4, C1, SE	0.041	(0.020)	<0.0005	0.177	(0.062)	<0.0005
Direction 5, C2, SE	0.114	(0.018)		0.274	(0.056)	
Direction 6, C2, SE	0.058	(0.022)		0.186	(0.068)	
Direction 7, C2, NE	0.099	(0.018)		0.295	(0.057)	

B. Time of Day, Referenced to 05:00-07:59 for Day and 23:00-23:59 for Night

Daytime Proportion ≤10 Lux				Nighttime Proportion ≤10 Lux			
Time of Day	β Coeff	St Error	p-value	Time of Night	β Coeff	St Error	p-value
08:00 - 10:59	-0.336	(0.008)		00:00 – 00:59	0.050	(0.016)	
11:00 – 13:59	-0.340	(0.008)		01:00 – 01:59	0.085	(0.016)	
14:00 – 16:59	-0.319	(0.008)	<0.0005	02:00 – 02:59	0.088	(0.016)	<0.0005
17:00 – 19:59	-0.197	(0.008)		03:00 – 03:59	0.087	(0.016)	
20:00 – 22:59	0.057	(0.008)		04:00 – 04:59	0.062	(0.016)	

Abbreviations: β Coeff, Beta-coefficient; C1, campus 1; C2, campus 2; COVID, SARS-CoV-2; Day 1 of ICU Admit, first 24 out of 48 hours of MICU admission; ICU, intensive care unit; Mechanical Vent, Mechanical Ventilation; NE, northeast; NW, northwest; Proportion ≤10, proportion of all readings less than or equal to 10 lux; SE, southeast; SOFA, Sequential Organ Failure Assessment; St Error, standard error.
